# Supplementary material for: Unsupervised manifold learning of collective behavior
Source: PLoS Comput Biol. 2021 Feb 12;17(2):e1007811. doi: 10.1371/journal.pcbi.1007811 (PMC7906460; doi:10.1371/journal.pcbi.1007811)
Supplement: S1 Text — (PDF) [file pcbi.1007811.s001.pdf]

Idealized Simulations of Bird Flocking

Let N agents be initiated at time 0 with position chosen uniformly at random on the torus $\mathbb{T} = S^1 \times S^1$, and with bearing chosen uniformly at random from S^1. Write $B_i(r) = \{j : \|p_i - p_j\| < r\}$ for the set of agents within a radius r of agent b_i. The dynamics of our flocking model are written as follows:

$$\theta_i(t+1) = \langle \theta_j(t) \rangle_{i,r} + \eta \tag{S1}$$

$$p_i(t+1) = p_i(t) + v(\cos(\theta_i(t)), \sin(\theta_i(t))) \tag{S2}$$

where $\langle \theta_j(t) \rangle_{i,r}$ is the mean bearing of the agents within $B_i(r)$ and η is a Gaussian noise with standard deviation $\epsilon > 0$. (Note that this mean bearing is calculated by finding the local mean velocity, then setting $\langle \theta_j(t) \rangle_{i,r}$ equal to its angle with the abscissa.) Thus each agent's position is updated by flying a distance v in the direction determined (up to the perturbation η) by the average of the directions of all other agents within r units of the focal agent. Allowing r to vary over time, transitions between flocking and disordered behavior can be observed. Fig 1 in the main text shows typical system states with $N = 800$ agents in the incoherent and coherent phases.

In the figures and analysis of the manuscript, the datasets are generated with $N = 500$, taking the noise to be $\epsilon = \pi/5$, velocity $v = 1/320$, with minimum and maximum values of the interaction radius given by $r_0 = 0.0065$ and $r_1 = 0.06$.

Empirical Data on Collective Behavior in Fish

The fish behavioral data was gathered by Tonstrøm et al. by recording video at 30 frames per second of a group of 300 golden shiners moving about a tank of shallow water. The water level was low enough to essentially limit the fish to planar motion. Image analysis was employed to locate the fish within each frame, then match them across frames when possible. This allowed the research team to associate a unique identifier with each fish while its trajectory was under observation.

These fish are highly social, and due to the difficulty of rendering individual fish when

they are packed tightly into a region, and the possible conflation of two separate fish swimming near one another, individual fish are often 'dropped' from the data set. When this occurs the identifier associated with that fish is retired, and the position and velocity data for that particular trajectory terminates. However, when the fish again exceeds the liminal level of the image analysis it is registered as a 'new' fish and given a new identifier. As a result, the data garnered from a typical frame records position and velocity information for only 60 - 80% of the members, and individuals are typically tracked for about 200 frames (6.7 seconds) before its image is 'lost' and its identifier removed from future data. The resulting data set consists of 100298 frames (about 56 minutes) of position and velocity data for the school. For the study in the manuscript we selected a 5000 frame subset containing roughly an equal number of frames devoted to each of the behaviors observed (frames 40001 to 45000). This enabled us to use the simple k-means clustering algorithm, which otherwise suffers when the subgroups to be identified have widely varying sizes. (This is because the classical k-means algorithm creates hyperspherical clusters, where the hyperspheres must be centered within the convex hull of the data and have equal radii. Smaller clusters necessitate a description via smaller hyperspheres. See [37].)

As discussed in [30], human observation of the schooling behavior identified three separate behavioral regimes for the fish: milling, swarming, and polarized (see S1 Fig). In the milling state, the fish rotate as a group, circulating about a fixed point; this is a highly coherent group state. While swarming, the fish trajectories are disordered, with very little discernible group alignment. In this state the school is stationary, though individual fish may be moving in an uncoordinated fashion. Last, the second coherent group structure is the polarized state, which is characterized by the group having a coherent direction of motion, and strong local alignment among the agents. As with the flocking model above, the variables of interest are the two-component vectors of fish position, p_i, and fish velocity, v_i.

Metrics

Let $x_i \in X$ be the set of observations for an agent (either a particle in the flocking simulation or a golden shiner found in the school) at a fixed time t, while $p_i(t)$, $\theta_i(t)$, and $v_i(t)$ are the ith agent's position, heading, and velocity, respectively. The first metric, $d^{(1)}$,

is simply the euclidean distance between the agents,

$$d^{(1)}(x_i, x_j; t) := \|p_i(t) - p_j(t)\|_2. \tag{S3}$$

For the flocking simulation, the second metric is the following

$$d^{(2)}(x_i, x_j; t) := \left(\sum_{k=0}^{30} g(k) \|v_i(t-k) - v_j(t-k)\|_2^2 \right)^{\frac{1}{2}} \tag{S4}$$

where $v_i(t) = v(\cos(\theta_i(t)), \sin(\theta_i(t))$ is the ith velocity at time t. The smoothing term $g(k)$ is proportional to $\exp\left(-k^2/30\right)$, but normalized so that $\sum_k g(k) = 1$. That is, $d^{(2)}$ is a gaussian smoothed time-average of the L^2 norm between velocities over the past 30 time steps.

For the fish, we consider a generalization of the previous expression for $d^{(2)}$. Rather than comparing the two fishes' heading time series at simultaneous moments, we allow one time series to be shifted into the past by a lag of up to $L = 60$ video frames, or two seconds. Define

$$\langle x_i, x_j \rangle_{L,t} := \min_{0 \le l \le L} \left(\sum_{k=0}^{30} g(k) \|v_i(t-k) - v_j(t-k-l)\|_2^2 \right)^{\frac{1}{2}},$$

where $g(k)$ is again a gaussian smoothing kernel as in the previous paragraph. Thus l represents a lag allowing us to compare not only concurrent velocity profiles over 30 steps, but also cases where fish i is mimicking a trajectory of fish j up to two seconds in the past. Note that $\langle x_i, x_j \rangle_{L,t} \neq \langle x_j, x_i \rangle_{L,t}$, as the righthand term calculates the distance between the trajectories under the assumption that j is following i. We define our distance as

$$d_L(x_i, x_j; t) := \min\{\langle x_i, x_j \rangle_{L,t},\ \langle x_j, x_i \rangle_{L,t}\}. \tag{S5}$$
