# Supplementary material for: Unsupervised manifold learning of collective behavior
Source: PLoS Comput Biol. 2021 Feb 12;17(2):e1007811. doi: 10.1371/journal.pcbi.1007811 (PMC7906460; doi:10.1371/journal.pcbi.1007811)
Supplement: S1 Table — The percentage of the frames of a given state (milling, polarized, etc.) which is found in each of our unsupervised groups (G1*, G2*, N) are displayed below the counts. (PDF) [file pcbi.1007811.s002.pdf]

	Milling	Swarming	Polarized	Transitional
G1*	1704<br>(100.0%)	6<br>(1.8%)	375<br>(26.4%)	606<br>(39.2%)
G2*	0<br>(0%)	106<br>(32.3%)	941<br>(66.2 %)	548<br>(35.5 %)
N	0<br>(0%)	216<br>(65.9%)	105<br>(7.4 %)	391<br>(25.3%)

S1 Table. Counts of classifications by our map alignment-based statistics (rows) and by the macro-scale variable classification (columns) for 5000 frames of fish movement data. The percentage of the frames of a given state (milling, polarized, etc.) which is found in each of our unsupervised groups ($G1^*$, $G2^*$, N) are displayed below the counts.
