# Supplementary material for: Unsupervised manifold learning of collective behavior
Source: PLoS Comput Biol. 2021 Feb 12;17(2):e1007811. doi: 10.1371/journal.pcbi.1007811 (PMC7906460; doi:10.1371/journal.pcbi.1007811)
Supplement: S2 Table — The 2-group clustering gave G1* and G2*, corresponding to frames of high and low macro-scale organization, respectively. The 3-group clustering gave G1, G2, and G3, corresponding to milling, α-polarized, and β-polarized behaviors, respectively. The percentages are with respect to all the frames in the entry’s column. (PDF) [file pcbi.1007811.s003.pdf]

	G1	G2	G3	N
G1*	1886 (99.95%)	803 (72.87%)	2 (0.15%)	0 (0.0%)
G2*	1 (0.05%)	299 (27.13%)	1295 (99.85 %)	0 (0.0 %)
N	0 (0.0%)	0 (0.0%)	0 (0.0 %)	712 (100.0%)

S2 Table. Comparison of the two k-means classifications for 5000 frames of fish movement data. The 2-group clustering gave G1* and G2*, corresponding to frames of high and low macro-scale organization, respectively. The 3-group clustering gave G1, G2, and G3, corresponding to milling, α-polarized, and β-polarized behaviors, respectively. The percentages are with respect to all the frames in the entry's column.
