# Supplementary figures and images for: Unsupervised manifold learning of collective behavior
Source: PLoS Comput Biol. 2021 Feb 12;17(2):e1007811. doi: 10.1371/journal.pcbi.1007811 (PMC7906460; doi:10.1371/journal.pcbi.1007811)

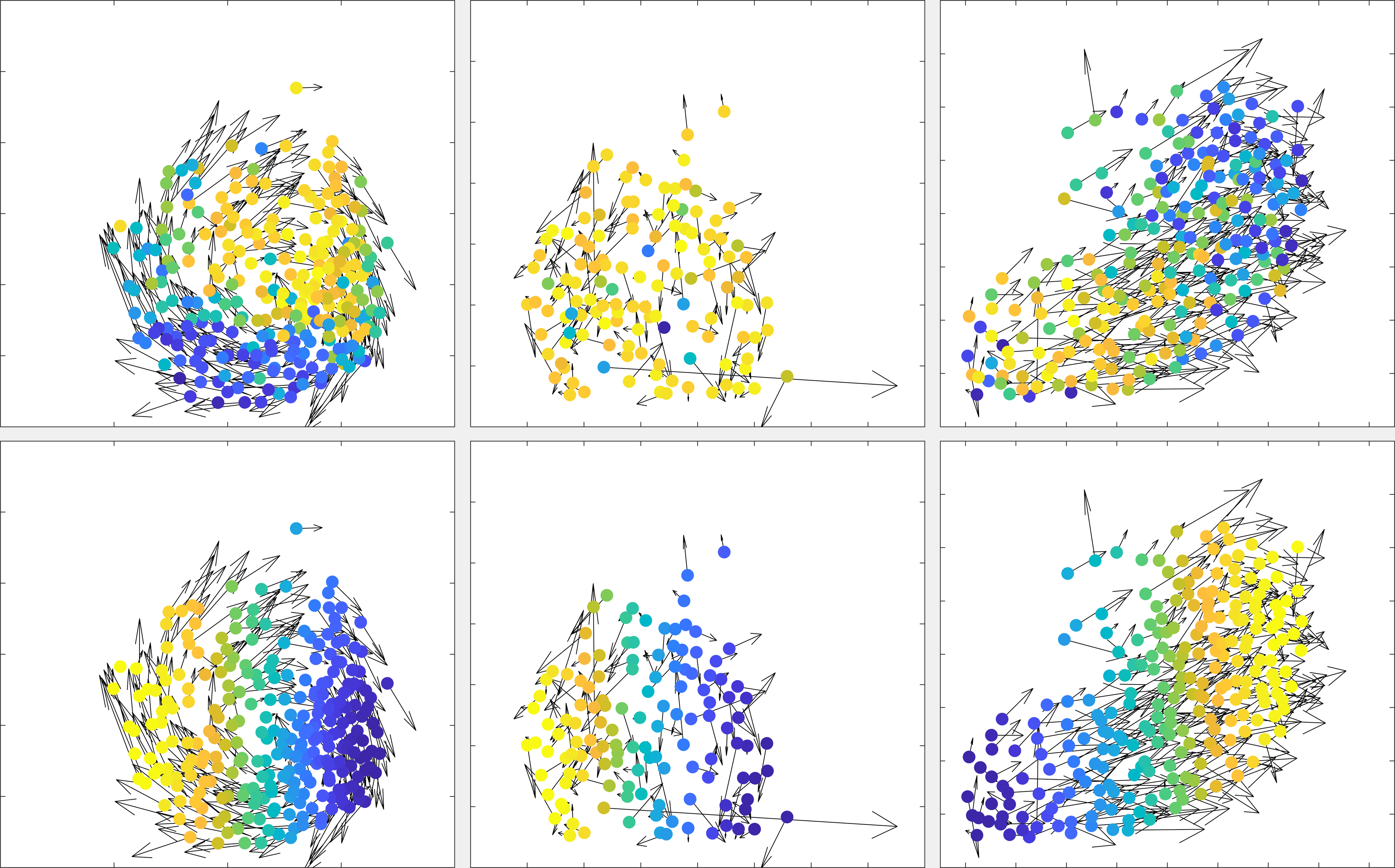

Supplement: S1 Fig — The emergent behaviors from left to right are milling, swarming, and polarized motion. (TIF) [file pcbi.1007811.s004.tif]
